# Encounters with patients from Somalia: experience among vocational trainees in Swedish general practice

**DOI:** 10.5116/ijme.51fc.dc05

**Published:** 2013-08-18

**Authors:** Kristian Svenberg, Bengt Mattsson, Margret Lepp

**Affiliations:** 1 Department of Primary Health Care, Sahlgrenska Academy, University of Gothenburg, Sweden; 2 Institute of Health and Care Sciences, Sahlgrenska Academy, University of Gothenburg, Sweden

**Keywords:** Experience, refugee, Somalia, transcultural encounter, vocational trainee, medical education

## Abstract

**Objectives:**

This study sought to describe how vocational trainees in general medical practice in Sweden experienced encounters with refugee patients from Somalia.

**Methods:**

Sixteen vocational trainees in general medical practice in Sweden were interviewed in focus groups. The interviews were transcribed verbatim and analyzed according to a phenomenographic approach.

**Results:**

Three categories with subcategories emerged. In the first category, “meeting the patient”, the family diversity among the patients was noted. Further, the informants noted that few patients presented psychiatric problems. In the second category, “obstacles in the encounter”, the vocational trainees noted difficulties in talking through an interpreter, who often seemed to have an extended dialogue with the patient. Obtaining a medical history was considered a challenge. The third category dealt with how to develop different strategies in the encounter.

**Conclusions:**

To improve the encounter with patients from Somalia and other minority groups, the importance of curiosity, trust and continuity of care should be discussed in medical education. Health care´s own ways of working and thinking in relation to matters of diversity must be observed in medical training.

## Introduction

Contemporary Europe and Sweden show increasing cultural diversity and heterogeneity.^1^^,^^2^ To health care systems, the associated vicissitudes pose new questions and demands, influencing both parties in the medical encounter. More and more, physicians in today’s Sweden have to relate to a new diversity and to patients with backgrounds and experience much different from their own. Demands for efficiency and meeting these patients impose contradictory pressures on physicians.^3^ The medical encounter is a complex event, involving individual and collective interplay in the light of existing medical and societal cultures. In the intimacy of the consulting room, the doctor has to handle an infinite variety of complaints and symptoms. The intricacies of this setting may be aggravated if communication is obstructed by language barriers and cultural discordance.^4^^,^^5^ To the patients, immigrants in particular, this situation involves a struggle for understanding, and this may shed light upon inherent power structures of the consultation.^6^^-^^8^Research covering the transcultural meeting is wide and complex, as is that covering the concept of culture in general.^9^^-^^12^ Physicians behave less affectively towards minority patients, hampering rapport-building and treatment outcomes.^13^ Minority patients, especially those not proficient in the majority language, seem less likely to receive empathic responses from physicians and obtain sufficient information, rendering mutuality in medical decision making thorny.^14^^-^^16^Medical education in Sweden regarding treatment of patients from other cultures has been reported as insufficient, and the role of the physician in culturally discordant meetings is not often openly addressed.^17^ In many Swedish medical curricula, as in Göteborg, the subject is often conspicuous by its absence. Medical education in Sweden has long adopted a patient-centered model for communicating, eliciting the patient’s thoughts and ideas regarding illness and symptoms.^18^^-^^20^ However, doctor’s thoughts and ideas regarding the patient have not received the same attention, a fact that is particularly valid for the transcultural encounter.With increasing numbers of refugees from war-torn Somalia, Swedish health care nowadays encounters patients from Somalia on a daily basis. During 2010, more than 6000 Somalis were granted political asylum in Sweden, constituting the largest asylum-seeking group followed by Afghanis and Iraqis.^21^ Approximately 40,000 Somali refugees today live in Sweden, having escaped the violent and chaotic situation in their homeland. Other large Somali refugee communities exist in England, Holland, USA, Canada and Australia, often with shattered family networks and with consequences of personal turmoil and strife.^22^ Research regarding these patients in the medical encounter is generally scarce. Internationally, exceedingly high expectations of health care have been reported among Somalis^23^^,^^24^ as well as frustration and perceived diminished quality of care.^7^^,^^25^^,^^26^ Reduced confidence in health care among Somali refugees and humiliation in contact with authorities in general have been reported.^27^^-^^29^ In Sweden, conceptions regarding health and illness among Somali refugees have been researched, as have perceptions of pain and matters regarding pregnancy, delivery and living with diabetes.^30^^-^^33^ Experience of Somali refugees and their encounters with Swedish health care have been studied. As a result of reduced confidence in Swedish health care, many Somalis seek medical advice and treatment in other countries.^34^To date, and to our knowledge, no studies have illuminated the experience of Swedish health care in encountering patients from Somalia. To fill this gap, the present study sought to describe how vocational trainees in general medical practice in Sweden experience encounters with patients from Somalia.

## Methods

### Study design

This study was conducted using a phenomenographic approach. Phenomenography aims to describe the qualitatively different ways a group of people make sense of, understand and experience a phenomenon in the world around them.^35^^,^^36^ In this study, the phenomenon was the experience among vocational trainees of their encounters with patients from Somalia.

### Participants

The study was conducted during spring 2010. The 16 participants (see Table 1) were all taking part in final training aiming at specialisation in general practice. Swedish medical education comprises 11 terms preceding graduation, followed by a two-year internship leading to authorization. Next, a five-year postgraduate training is followed, leading to a medical specialisation. To qualify for the present study, the participants were required to have present or recent experience of seeing patients from Somalia. The participants were active in three large Swedish cities – Stockholm, Göteborg and Malmö - and were contacted through key persons at the universities in each city, using e-mail and phone. Before the interviews, information concerning the study was distributed to the trainees and preparatory meetings were organized together with the researchers and the participants. Finally, four groups were formed, each with on average four to six participants. Seven were male and nine female, with an average age of 37. Nine participants had their basic medical education in Sweden and seven in Greece, Holland, Hungary, Germany, Russia, Iran or the Czech Republic. Approximately one-third of physicians today working in Sweden had their basic medical education abroad (Swedish National Board of Health and Welfare, personal communication 2011).

**Table 1:** Profile of the participants, 16 vocational trainees in General Practice

Variable		n
Age		
	25-30	2
	31-35	4
	36-40	5
	41-46	5
Gender		
	Male	7
	Female	9
Years of experience in seeing patients from Somalia		
	1-2	2
	3-4	11
	5	3
Basic medical education in		
	Sweden	9
	EU	5
	Outside EU	2

### Data collection

In phenomenography, interviews are a common means of data collection. In this study, four focus-group interviews were conducted with four to six participants at a time. The essence of a focus-group interview is the active participation of the interviewees, leading to a productive conversation, arousing association and stimulating a flow of ideas among the participants.^37^ No specific interview guideline was used. An open question was put at the introduction of the interviews. This question was: “Could you tell us about your experience of seeing patients from Somalia?” To stimulate the discussion, follow-up questions were put to the physicians. The interviews were informal and conversational and the participants overall were active and enthusiastic. Each focus group interview lasted on average two hours. The first author was aided by an observer who was part of the research team and all the interviews were tape-recorded.

### Analysis

The interviews were transcribed verbatim by the first author and analysed according to the phenomenographic research approach.^38^^,^^39^ The interview text then underwent an interpretive process aimed at organising the text through a course of deconstruction and reassembly. Repeated reading gave a familiarisation with the material. Then, the focus was sharpened to detect the various conceptions of the participants, the variations among them and how these differed. Through further readings, three categories with subcategories emerged, describing the variation of conceptions within the different domains of the data material. This constituted the ‘outcome space’.^38^

### Trustworthiness

In qualitative research it should be possible to follow the researchers’ rationale throughout the study. To obtain a high level of trustworthiness, the categories must be thorough and represent the participants’ perceptions, not simply being a construction of the researcher.^40^ The quotes given in this study are intended to facilitate the readers’ evaluation of the trustworthiness of the analysis. To warrant the data analysis, a group of co-examiners tested the results. The study was approved by the Research Ethics Committee, University of Gothenburg. Written and verbal information was given to the participants prior to the interviews. The study was performed according to general ethical procedures such as voluntariness and possibility to discontinue the participation at any time.

## Results

**Figure 1:**
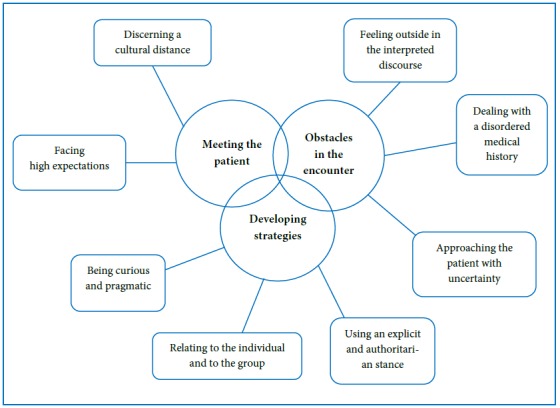
Categories (bold) and subcategories of conceptions of encounters with the patients

Three categories – meeting the patient, obstacles in the encounter and developing strategies, emerged in the analysis. To each category subcategories surfaced, describing the participants´ various conceptions of their encounters with their patients (see Figure 1).

### Meeting the patient

This category dealt with meeting the patient as a person, and consisted of two subcategories: facing high expectations and discerning a cultural distance.

#### Facing high expectations

Talking to their patients, the physicians experienced a strong focus on bodily symptoms. Expectations on the physician were high, as confirmed by several statements. Patients expected an explanation and a treatment. A doctor asking too many questions may be considered incompetent. This puts the doctor to the test:

“Well, the more you ask….you are expected to be able to say something with a minimal amount of information. The patient enters the room with a certain aura which I am supposed to read, exactly what is the matter with them. They don’t have to utter a word, and I’m supposed to make a diagnosis just like that!” *(informant nr 15, female)*

Mind-body dichotomy was considered by the informants to be strong among the Somali patients. As an example, a female trainee said that if she tried to explain diffuse symptoms as caused by ‘stress’, the patients didn’t seem to accept this, generally considering the cause to be physical. Abdominal pain was often considered to be caused by ‘renal problems’, the patient frequently having a fixed somatic explanation for the suffering.

“Many Somalis come with abdominal problems, bellyache, and they usually blame the kidneys. I have sent them for different investigations without any results….” *(informant nr 9, male)*

#### Discerning a cultural distance

This subcategory contained conceptions of feelings of remoteness and alienation, displaying difficulties in understanding due to an emotional space in the encounter:

“There is a distance between me and the patient. We never get that close, they don’t show me any special feelings. They tell me about their pains and bodily symptoms but they never come emotionally close, they never cry.” *(informant nr 5, male)*

According to the statements, suffering in the form of pain and diffuse bodily symptoms seemed frequent among the patients. However, mental problems were considered by the majority of the participants to be virtually non-existent as a reason for attending the physician. Explanations offered for this were strong social networks and family bonds, and ideas about mental disease being taboo and risking stigmatization.

“I never had a patient who came to me for lack of sleep, anxiety or depression. Usually they come for headache and pain in the body.” *(informant nr 6, female)*

Some statements stressed the existence of mental problems among the patients, though in a more indirect way:

“Anxiety is rather common among women, if you penetrate it a bit further*…” (informant nr 12, female****)***

A number of statements referred to many patients as unyielding and happy, displaying pride and integrity. Women, usually accompanied by children and chaperones, seemed to attend health care more frequently than men, who appeared absent in one way or another, rarely seeing the physician. It was strongly believed that the children were easily and rewardingly examined.Often, patients’ family bonds and relationships seemed confusing. Extended family structures appeared to be common:

“If there are three or more persons in the room, I usually ask ‘are you the sister?’ or ‘are you the mother?’ I want to know who they are, I don’t want to hear at the end of the meeting that they do not know each other. It just is good for me to know who they are.” *(informant nr 10, male)*

**Table:** 

### Obstacles in the encounter

The second category dealt with conceptions related to obstacles in the encounter. It consisted of three subcategories: Feeling outside in the interpreted discourse, Dealing with a disordered medical history and approaching the patient with uncertainty.

#### Feeling outside in the interpreted discourse

The use of an interpreter in the clinical encounter was a common feature when seeing the patients, resulting in a complex triadic meeting. Unanimously, communicating through an interpreter was regarded as a difficulty:

“I think they end up in a private conversation, the interpreter and the patient. So I don’t really grasp what’s going on. I put a question and they talk for two minutes and I don’t understand what they say and why they talk for so long.” *(informant nr 2, male)*

Some statements included explanations of the verbose manner of many interpreters as an attempt to explain the questions to the patient in a cultural context.

#### Dealing with a disordered medical history

The statements included various obstacles in obtaining a good medical history as well as getting ‘compliance’ from the patient. Rapport was often difficult to achieve, especially in chronic diseases such as diabetes.

“They sit in front of me talking about many strange symptoms and feelings. And I ask ‘are you worried for something special?’ and I don’t get anything back from them. It seems as if our behaviours don’t fit…” *(informant nr 12, male)*

#### Approaching the patient with uncertainty

Facial expressions revealing non-verbal cues among many patients seemed to be of rare occurrence, according to the statements. As a result, there were difficulties in interpreting the patient’s communicative behaviour, hampering paralinguistic understanding.

“I can’t see if they are sorry, or interested or curious. The facial expression is so stable in a way, I have to interpret the words instead….” *(informant nr 1, female)*

There was a diversity of modes of greeting among the patients, the women being prone not to shake hands with male trainees. To try to avoid this conflict, the following strategy was elaborated by one male informant:

“I try to make it easy. I just put my hand over my chest and say ‘salaam alaicum (peace be with you)’. It always works*!” (informant nr 14, male)*

### Developing strategies

This category covered statements on how to adopt different strategies to handle the consultation. It consists of three subcategories: Using an explicit and authoritarian stance, Relating to the individual and to the group and Being curious and pragmatic.

#### Using an explicit and authoritarian stance

According to some statements, information should be exchanged in plain and explicit terms. Too much discussing was considered as promoting obscurity and wrecking tight time schedules.

“I talk to Somalis in a telegraphic sort of way. No long discussions, no way. Just bang, bang. Otherwise you’re *lost.” (informant nr 13, male)*

#### Relating to the individual and to the group

In some statements, too much ‘fishing’ about the patient’s background and ethnic origin seemed embarrassing. Referring to their own experience as immigrants, some informants expressed doubts about this approach:

“I seldom ask about their origin. That’s because I’m an immigrant myself and I feel that I would discriminate the other by asking “where do you come from?” To me, they are human beings just like anybody else, and I want other people to look at me in the same way.” *(informant nr 4, female)*

Paying attention to the individual had priority according to a majority of the statements. Collective experience could be noted, but with caution.

“To start with, I see the individual and then I can put the individual in relation to the group. Only seeing the individual could be a disadvantage, you can lose certain cues from the patient, certain expectations from the patient who´s not familiar to you.” *(informant nr 6, male)*

#### Being curious and pragmatic

Here, a majority of the statements expressed a curious and affectionate position in the medical encounter, exploring the patient’s social background and personal history. Curiosity was regarded as fostering understanding:

“If the situation is complicated, I usually ask about their background. I want to negotiate a curious attitude, how they grew up and so on, it all depends on what kind of problem they come up with.” *(informant nr 8, female)*

## Discussion

The findings suggest a variety of conceptions regarding personal traits among the patients, obstacles in the encounter and developing strategies in the meeting.In meeting the patient, the participants noted high expectations on health care among the patients. The physician is often expected to make a diagnosis based on a minimal amount of information, an experience supported by research abroad.^23^^,^^25^ Ambiguities appear in the conceptions of many Somali patients, women in particular, as happy and strong. Though many consider this a fact, some demonstrate uncertainty as to whether this is a façade, veiling grief and trouble. Research often supports this stance, indicating that Somalis in exile suffer poor mental health due to lack of social support and loss of family networks as well as chronic sorrow and previous experience of war trauma.^25^^,^^41^^-^^43^Presenting a strong and resilient attitude could well function as a coping strategy in a trying situation, as well as indicating a way of handling distress. To the health care provider, this paradox may not be obvious. The appearance of the other as ‘strong and happy’ may unconsciously function as an excuse in underestimating non-salient cues of mental distress, particularly in today´s rigid clinical time-schedules. To the medical trainees in this study, the findings suggested the need for medical education to pay attention to the meeting with “the other”, where the needs and expectations of minority patients could be discussed.Obstacles in the encounter dealt with relational aspects of the medical meeting. Language is of a central concern. Being the object of a considerable body of research, working with an interpreter is a challenge.^14^^,^^44^^-^^47^ This is to be considered when trying to understand the difficulties for both physicians and patients in the process of creating meaning and understanding. To medical students and trainees, this calls for increased vigilance and alternative strategies in observing the patient’s paralinguistic cues and body language. It has been demonstrated that medical students early on in their education elicit patients’ narratives in a deeper sense than during later curricula when they seem to be more occupied by the ‘learned expertise’ of biomedicine.^48^^-^^50^ It is worth considering whether the strong emphasis some of the present participants put on the importance of a good medical history reduces their possibilities of getting closer to the patient.It has been pointed out that minority and refugee patients are less verbally expressive and even less affective during the consultation than patients sharing the same ethnic background with their physician.^4^ The faces of many patients were experienced as bland by physicians in this study, having difficulties in reading their nonverbal expressions. Referring to Levinas, it has been argued that the face in its essence transcends history and culture bringing to the fore the obligations towards “the Other” irrespective of existing categorizations and norms.^51^ Thus, the distance often experienced in relation to the patients in this study could mean the starting point towards new understanding, putting both the physician and the patient to the test in achieving a shared understanding in the medical meeting. How symptoms are communicated by some of these patients should be observed and discussed in medical education. This might also lead to new and different meanings that challenge the biomedical framework.^52^In the last category, developing strategies, the participants elaborated some views on how to approach their patients. In research on how physicians meet and treat refugee patients a ‘technical strategy’ versus a ‘human interest strategy’ has been stressed, bearing some resemblance to the strategies mentioned in this study.^5^ Others noted that physicians meeting immigrant patients oscillated between insistence on patient adaptation and physician adaptation as well as negotiation, depending on the topics raised. Generally they had no framework for eliciting information concerning the patient’s culture.^53^ In a Swedish study, GP-s used the same approach when seeing immigrant patients as when meeting native-born Swedes.^54^The curious and pragmatic attitude, demonstrated by some informants, is more in line with widening the internal medical context, allowing entrance to parts of the patient’s life-world. Integrating principles of rationality and relationality could encourage a basis for sharing meanings and narratives in ‘cross-cultural’ settings.^15^ An authoritarian strategy, as represented by some statements in this study, could be replaced by proximity and emotion.^55^ An interest in the person behind the symptom and “cultural label” might foster mutual understanding.^12^ Here, medical education has a responsibility to counteract the objectification of patients. A balance between “difference”, attention to the student and attention to power relations could be established.^56^ Structural factors in health care may be counterproductive in the building of trust through continuity, a fundamental prerequisite of medical practice and of general practice in particular.^57^^-^^59^ Indeed, encounters with refugees and patients from minority groups, often needing interpreters, are especially vulnerable to fragmentation and discontinuity.^60^

### Limitations

In the capacity of senior GP, the main interviewer might have impeded spontaneity among the participants. The other two authors, who took turns in participating in the focus groups, were both senior academicians. This also could have negatively influenced the creativity of the participants. Possibly, the responses might have been different if the interviewers had been external researchers.

### Reflections on method

Ideally, focus-group interviews give the advantage of a collective flow of ideas and viewpoints among the participants. However, this may occur at the expense of individual standpoints, trying to express occurrences of emotional and personal meaning. A single strong voice may tend to dominate the group, and this should be constantly remembered and handled by the interviewer, creating opportunities for everybody’s voice to be heard. As for the analysis of the empirical data, phenomenography, rather than being a methodology is more of an analytic and explorative approach to qualitative research.^61^The vocational trainees in this study discussed their meetings with their ‘Somali patients’. Ethnic categorization, being a sensitive matter, may cloud internal variation and exaggerate homogeneity within groups.^62^ The expression “Somali patients” risks reinforcing the difference already construed in the discourse of this particular refugee group in Swedish society. With this in mind, the term should not be regarded as a fixed classification. Notwithstanding the heterogeneity and disparity in this group of patients, there could be lessons to be learnt from meeting minority-group patients in general.

## Conclusion

This study has explored how vocational trainees in Swedish general medical practice experienced their encounters with patients from Somalia. Predicaments in achieving understanding seemed common, originating in a complex web of factors confusing the interpretive situation. Eliciting the complexity of the doctor’s position, especially when seeing refugee patients, could be a starting point towards a more democratic approach to bringing forth the intricacies of communication in the transcultural meeting.Acknowledging uncertainty and shifting from a spotlight on cultural differences towards a focus on the patient as an individual might help to develop a constructive approach when wrestling with diversity.^63^ In medical education, these subjects need urgent attention to facilitate the outcome of culturally discordant consultations.

### Meaning and implications of the findings for medical education

1. Meeting the patient: In medical education, the individual aspects of each patient should be stressed while not forgetting the collective experiences of a particular group of patients. The importance of the patient´s cultural background and its influences on what they expect of health care should be noted. At the same time, the culture of bio-medicine needs to observe its own effect on how to approach cultural diversity in health care. Attitudes and predefined contexts regarding norms and the power balance of the medical meeting should be highlighted.
2. Obstacles in the encounter: How to work with a professional interpreter should be taught in special courses. Techniques and pitfalls of the medical discourse via an interpreter should be highlighted.
3. Developing strategies: How to use curiosity and continuity of care in the building of trust in the medical encounter – especially when meeting patients from ethnic minorities - should be discussed and learnt.

### Conflict of Interest

The authors declare that they have no conflict of interest.
